# Mechanisms of Venoarteriolar Reflex in Type 2 Diabetes with or without Peripheral Neuropathy

**DOI:** 10.3390/biology10040333

**Published:** 2021-04-15

**Authors:** Cécile Reynès, Antonia Perez-Martin, Houda Ennaifer, Henrique Silva, Yannick Knapp, Agnès Vinet

**Affiliations:** 1 STAPS Department, Avignon Université, LAPEC EA4278, 84000 Avignon, France; cecile.reynes@gmail.com; 2 Service Exploration et Médecine Vasculaire, Hôpital Universitaire de Nîmes, IDESP Université de Montpellier, 34000 Montpellier, France; antonia.perez.martin@chu-nimes.fr; 3 Service Endocrinologie et Maladies Métaboliques, Centre Hospitalier Henri Duffaut, 84000 Avignon, France; ennaifer.houda@ch-avignon.fr; 4 Research Institute for Medicines (iMed.ULisboa), Faculdade de Farmácia, Universidade de Lisboa, Av. Prof. Gama Pinto, 1649-003 Lisbon, Portugal; henrique.silva@campus.ul.pt; 5 Department of Pharmacy, Pharmacology and Health Technologies, Faculdade de Farmácia, Universidade de Lisboa, Av. Prof. Gama Pinto, 1649-003 Lisbon, Portugal

**Keywords:** type 2 diabetes, peripheral neuropathy, laser-Doppler flowmetry, microcirculation, venoarteriolar reflex

## Abstract

**Simple Summary:**

Postural changes induce arteriolar vasoconstriction in response to an increase in venous pressure in the limbs known as the venoarteriolar reflex (VAR). Previous studies reported that skin blood perfusion is impaired during the VAR in persons with type 2 diabetes mellitus (T2DM) with or without peripheral neuropathy, compared to control subjects. The aim of this study is to investigate the underlying mechanisms of the VAR in T2DM, with and without peripheral neuropathy. This study provides evidence that confirmed neuropathy alters the VAR by an alteration of the neurogenic response to leg dependency.

**Abstract:**

The aim of this study is to investigate the underlying mechanisms of the venoarteriolar reflex (VAR) in type 2 diabetes mellitus (T2DM), with and without peripheral neuropathy. Laser Doppler flowmetry (LDF) recordings were performed on the medial malleus and dorsal foot skin, before and during leg dependency in healthy controls, in persons with obesity, in those with T2DM, in those with T2DM and subclinical neuropathy, and in those with T2DM and confirmed neuropathy. LDF recordings were analyzed with the wavelet transform to evaluate the mechanisms controlling the flowmotion (i.e., endothelial nitric oxide-independent and -dependent, neurogenic, myogenic, respiratory and cardiac mechanisms). Skin blood perfusion decreased throughout leg dependency at both sites. The decrease was blunted in persons with confirmed neuropathy compared to those with T2DM alone and the controls. During leg dependency, total spectral power increased in all groups compared to rest. The relative contribution of the endothelial bands increased and of the myogenic band decreased, without differences between groups. Neurogenic contribution decreased in controls, in persons with obesity and in those with T2DM, whereas it increased in subclinical- and confirmed neuropathy. In conclusion, this study provides evidence that confirmed diabetic neuropathy alters the VAR through the neurogenic response to leg dependency.

## 1. Introduction

Postural changes induce arteriolar vasoconstriction in the limbs, in response to an increase in transmural venous pressure, known as the venoarteriolar reflex (VAR). This acute vascular adaptation to orthostatism limits the increase of hydrostatic pressure in the peripheral capillaries, acting as an important edema prevention mechanism. It has been shown that the VAR is under local control, mediated by a change in the myogenic tone and by the release of transmitters from non α-adrenergic nerve fibers, rather than by local sympathetic control [1,2]. Recently, it was found that endothelial nitric oxide (NO)-independent mediators, as well as Ca^2+^ and voltage-gated K^+^ channels contribute to the VAR [3,4]. However, the underlying mechanisms of this vascular response are still debated.

In type 2 diabetes mellitus (T2DM), the VAR may be altered and previous studies reported an increase in skin blood perfusion during the VAR in these patients compared to the control subjects. The presence of typical peripheral neuropathy, one of the most common complications of T2DM, worsened VAR impairment, and was associated with an exacerbated microvascular dysfunction [5]. The reduction in the vasoconstrictive response was demonstrated in the standing position [6,7], sitting up [8,9,10] and in limb-dependent protocols [11,12]. The loss of the postural regulation of skin blood flow and raised capillary pressure may have important consequences for the diabetic foot, due to an increase in edema formation. These abnormalities may initiate microvascular damage and may contribute to the development of foot complications [9]. Nevertheless, the cause of VAR disturbance in T2DM is still unclear. 

Previous studies used laser Doppler flowmeter (LDF) recordings, however, without considering the underlying mechanisms of skin blood perfusion. Flowmotion, obtained by spectral analysis of LDF signals and periodic oscillations, allows to look into the assessment of mechanisms of perfusion regulation [13]. Within the available frequency spectrum, six representative frequency bands are related to the cardiac, respiratory, myogenic, neurogenic, endothelial NO-dependent, and NO-independent components; encompassing central and local control mechanisms [14]. Thus, the use of sensitive signal-processing tools such as the wavelet analysis of the LDF signal during the VAR could provide data to explain the mechanistic alterations in diabetic peripheral neuropathy.

Accordingly, the aim of this study was to compare skin blood perfusion and flowmotion during the VAR response to leg dependency (i.e., lowering the limb from heart level to 30 cm below heart level) in four groups: control subjects, obese patients and T2DM patients, with or without neuropathy.

## 2. Materials and Methods

### 2.1. Participants

The NEUROMICRO trial (NCT03847779) is a clinical study designed to examine flowmotion following different stimuli in persons with T2DM. Eighty-two participants (38 females, 44 males) aged 40–72 years were included in this study after providing their informed written consent. Among them, 44 were T2DM patients, including 15 without peripheral neuropathy, 13 with subclinical peripheral neuropathy, and 16 with confirmed peripheral neuropathy. Of the other 38 participants, 22 were obese patients (matched with diabetic group for body mass index), while the remaining 16 subjects were considered healthy with normal-weight and were matched for age and sex to form the control group. All participants without diabetes (i.e., those with obesity and the healthy controls) were normotensive, were not using any medications, and had no evidence of cardiopulmonary disease. Patients with T2DM who had a history of ulceration, a toe-brachial index under 0.7 (indicating peripheral vascular disease [15]), or a history of using of vasoactive medications (e.g., calcium channel blockers) were excluded. In order to implement a real-life scenario in this study, patients maintained their quotidian medication (e.g., for high blood pressure, dyslipidemia, glycemic control or pain treatments) throughout the study (Table 1). 

All participants with T2DM were also screened for retinopathy, nephropathy, cardiac autonomic neuropathy. Retinopathy was evaluated by dilated fundus examination. Nephropathy was defined as a glomerular filtration rate under 90 mL/min/1.73 m^2^ according to the Chronic Kidney Disease Epidemiology Collaboration equation [16]. Cardiac autonomic neuropathy was evaluated with the three Ewing Tests: (1) The heart rate response to deep breathing (E/I ratio); (2) the heart rate response to standing (30 s/15 s ratio); and (3) sympathetic neural function via the blood pressure response to standing. For each of these three tests, a score of 0 was assigned for normal, 0.5 for limit, and 1 for abnormal results. Patients with Ewing score ≥1 were considered as having CAN and patients who had Ewing score less than 1 were not.

Peripheral neuropathy was detected by a Neuropathy Symptom Score questionnaire >3 [17]. Briefly, the presence of chronic, symmetrical, length-dependent sensorimotor polyneuropathy was based on alteration of large and/or small nerve fibers. Large nerve fibers were tested via sural nerve conduction using a DPNCheck device (Neurometrix, Waltham, MA). Abnormal nerve conduction was considered when an amplitude of ≤5 µV or a velocity of ≤41 m/s was reached [18]. An alteration of the small nerve fibers was considered when a score under three for hot or cold sensibility was obtained or if a thermal pain threshold was not reached between 35 and 45 °C when being assessed with NerveCheck (Phi Med Europe S.L., Barcelona, Spain). In accordance with the Toronto Consensus [19], peripheral neuropathy was confirmed when signs and symptoms were detected. In absence of symptoms, but in presence of signs, peripheral neuropathy was defined as subclinical. 

### 2.2. Protocol

All participants were instructed to present to their assessment at least two hours fasted and to abstain from caffeine and smoking. They were also instructed to avoid strenuous physical activity in the 24 h prior to their assessment. Blood samples were collected for HbA1C assessment in all participants except for the healthy controls.

All microvascular assessments were performed in a quiet, temperature-controlled room (22–25 °C) with minimal external disturbances, while the participant remained in a supine position. Skin blood perfusion was assessed using a laser Doppler flowmeter system (Periflux 5000, Perimed, Sweden) coupled with two thermostatic probes with a wavelength of 780 nm and an effective surface of 0.95 cm^2^ (PF 481, Perimed, Stockholm, Sweden). On intact skin of the right foot, one probe was placed under medial malleolus and the other on dorsal surface of the foot between the heads of the second and third metatarsals (Supplemental Data). Based on the Doppler principle, the probe emits and detects light partially backscattered in the tissues by moving blood cells, causing a change in frequency. The proportion of non-shifted to shifted light is related to the number of moving cells and the velocity of these cells, and is a characteristic of tissue perfusion. The typical depth of the measurement is 1–1.5 mm, evaluating non-nutritive skin blood perfusion. LDF recorded continuously at frequency of 32 Hz using an interfaced computer with data acquisition software (Perisoft, Perimed, Stockholm, Sweden) and skin blood perfusion were expressed as conventional perfusion units (PU). 

Prior to the start of the skin blood perfusion assessment, the feet were immobilized with a vacuum cushion. After an acclimatization period of 20 min, basal skin blood perfusion was recorded for 15 min in a supine position with both legs resting fully extended on the bed maintained at heart level. Participants were then asked to lower their right foot off the bed by flexion of the knee below the heart level, while the left foot kept horizontal still lying in a supine position. The right foot remained in this lower position five minutes. Brachial artery blood pressure was monitored at the beginning and at the end of basal period, and after the lowering foot period using a digital sphygmomanometer (OMRON 907, Omron Healthcare, Kyoto, Japan).

### 2.3. Data Analysis

**Figure 1:**
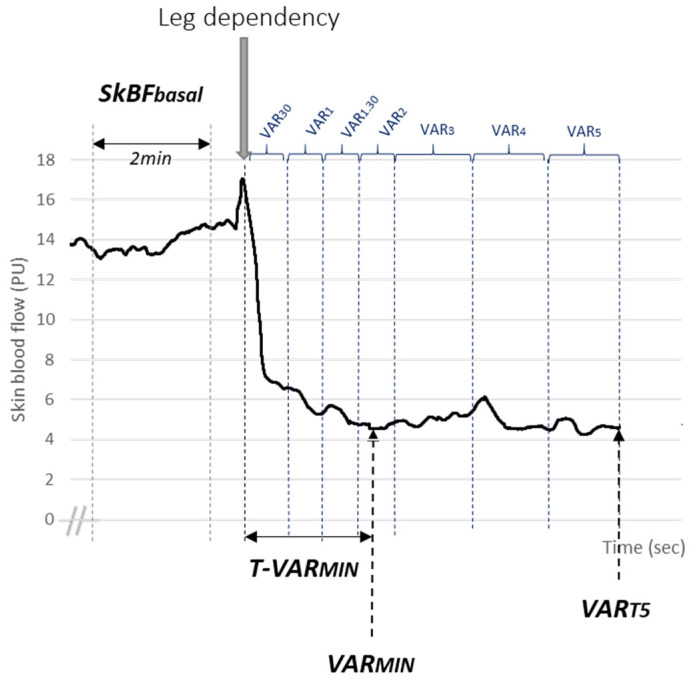
Representation of analyzed parameters. SkBF, skin blood flow.

An LDF recording was considered acceptable when there was a stable curve in the total backscattering signal. Basal skin blood perfusion is the most stable two minutes into the resting period. Indices of the VAR were considered first as kinetics, with changes in skin blood perfusion averaged every 30 s during the two first minutes after postural change, and every minute thereafter until the fifth minute (i.e., VAR_30_, VAR_1_, VAR_1.30_, VAR_2_, VAR_3_, VAR_4_ and VAR_5_). Second, after averaging skin blood perfusion every 20 s, the minimal perfusion during the leg dependency period (VAR_MIN_) and the time to reach VAR_MIN_ (T-VAR_MIN_), as well as the percentage reduction from rest (VAR_MIN_%) were assessed (Figure 1).

Continuous wavelet transforms based on early work by Bracic and Stefanovska [20], as well as subsequent research [13,14,21,22], were computed from the valid temporal data across the full length of the recording [23], which lasted 45 min. Spectral analysis was performed for 15 min from the end of the acclimatization period and, immediately, for another five minutes from the beginning of the leg dependency period. As a consequence of this procedure, data were not impacted by the cone of influence exerted by the wavelet transform [21]. The time-averaged spectrums were split into the following six frequency bands [24]: Band VI, Endothelial NO-independent mechanisms [0.005–0.0095 Hz]; Band V, Endothelial NO-dependent mechanisms [0.0095–0.021 Hz]; Band IV, Neurogenic mechanisms [0.021–0.052 Hz]; Band III, Myogenic mechanisms [0.052–0.145 Hz]; Band II, Respiratory mechanisms [0.145–0.6 Hz]; and Band I, Cardiac mechanisms [0.6–1.6 Hz]. Finally, the absolute median value of the total spectral power and of each band was computed; and the relative contribution (i.e., the median level of a particular frequency band divided by the median level of the total spectral power) was calculated for the rest and recovery periods across both skin sites.

### 2.4. Statistical Analysis

All statistical analyses were performed using SPSS^®^ (version 22.0, IBM Corp, Armonk, NY, USA). Variables were expressed as mean ± standard error of the mean. A general Linear Model was used to compare variables between groups and a linear mixed model was used for the kinetics analysis, as well as the comparison between the rest and leg dependency period. In the event of a significant group interaction, pairwise comparisons with Bonferoni post-hoc tests were used. According to the results of the correlation matrix between skin blood perfusion and potential cofactors, the general linear model and the linear mixed model were adjusted to skin temperature or to basal skin blood perfusion and/or age. A *p* < 0.05 was considered statistically significant.

## 3. Results

### 3.1. Participant Characteristics

The clinical characteristics of the participants are reported in Table 1. All groups were comparable in terms of sex and blood pressure with well-controlled values. Body mass index did not differ between those with obesity or T2DM, or those with T2DM and subclinical or confirmed neuropathy. As expected, however, body mass index was lower in the controls (*p* < 0.001). The group with confirmed neuropathy was older than those with obesity and those with T2DM alone. The duration of T2DM was not significantly different between the three groups with diabetes. As expected, HbA1c levels were higher in those with T2DM versus those with obesity (*p* < 0.001). The proportion of microvascular complications was not different between the groups with T2DM.

### 3.2. Microvascular Assessments

There was no change in heart rate and blood pressure during the VAR, whatever the group. Microvascular data for the medial malleus at rest and during the VAR are reported in Table 2. There was no difference in basal skin blood perfusion between groups in the supine position. VAR_MIN_ expressed in either PU or percentage, was not different between groups. Same results were obtained on dorsal surface of the foot (data not shown). All results were preserved after model adjustment to basal skin temperature, basal skin blood perfusion or age.

**Figure 2:**
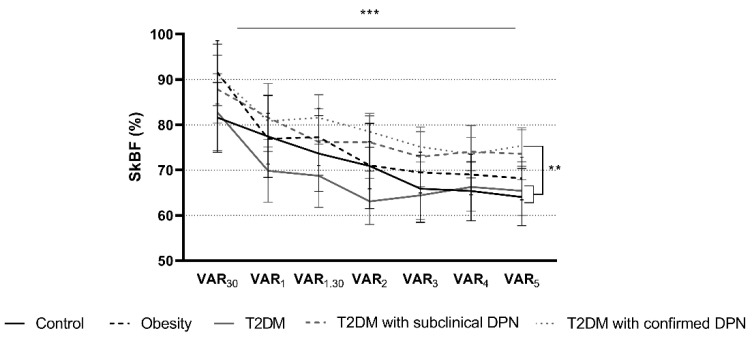
Skin blood perfusion kinetics during leg dependency at the medial malleus in each group. *** Time effect *p* < 0.001; ** Group effect: confirmed-DPN vs. controls and T2DM *p* < 0.01; interaction *p* = 1. DPN, diabetic peripheral neuropathy; T2DM, type 2 diabetes mellitus; SkBF, skin blood flow.

Regardless of the skin site and the group, skin blood perfusion at VAR_30_ was lower than basal skin blood perfusion. Skin blood perfusion kinetics expressed as a percentage of basal skin blood perfusion demonstrated a significant decrease throughout the leg dependency period (*p* < 0.001; Figure 2 and Figure S1) at both skin sites. At the medial malleus, a group effect was found with a blunted decrease in skin blood perfusion in those with confirmed neuropathy compared to controls and those with T2DM (*p* < 0.01). After adjustment for basal skin temperature and/or age, this blunted decrease remained across both skin sites. 

### 3.3. Spectral Analysis

At rest, irrespective of the skin site, there was no difference between the groups for total spectral power or for each frequency band. At the dorsal foot, the relative contribution of the respiratory band was lower in participants with obesity than in those with subclinical neuropathy or confirmed neuropathy (respectively 6.44 ± 0.85, 15.14 ± 4.82, and 14.02 ± 2.54%; *p* < 0.05). At the medial malleus, the relative contribution of the cardiac band was lower in those with obesity and confirmed neuropathy compared to controls (*p* < 0.05). All other bands were similar between each group.

After leg dependency, the total spectral power did not change significantly in the dorsal foot, whereas, it increased in the medial malleus without difference between groups. This increase was supported by a significant increase in the absolute values of the two endothelial and neurogenic bands, whatever the skin site (*p* < 0.01).

**Figure 3:**
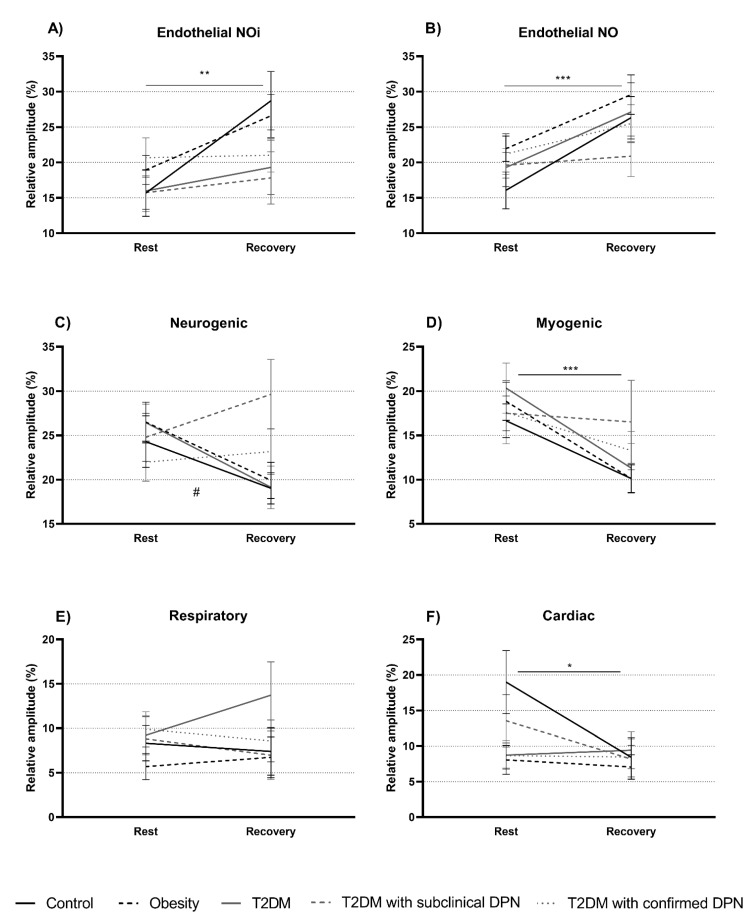
The relative contribution of each component at rest and during the leg dependency period at the medial malleus: endothelial NO-independent (**A**), endothelial NO-dependent (**B**), neurogenic (**C**), myogenic (**D**), respiratory (**E**), and cardiac (**F**) band. *** Time effect *p* < 0.001; ** Time effect *p* < 0.01; * Time effect *p* < 0.05; ^#^ Interaction Time × Group *p* = 0.07. DPN denotes diabetic peripheral neuropathy; T2DM, type 2 diabetes mellitus.

The relative contribution of the endothelial bands increased significantly irrespective of the skin site without difference between groups (Figure 3A,B and Figure S2). Myogenic and cardiac contributions decreased significantly at both skin sites without difference between groups (Figure 3D,F and Figure S2). At the dorsal foot, there was no time or group effect in the neurogenic band, but there was a decrease in the respiratory band across all groups, with the greatest decrease occurring in those with T2DM (Figure S2; *p* < 0.05). At the medial malleus, there was no change in the respiratory band (Figure 3E). There was a slight group by time interaction in the neurogenic band with an increase in those with subclinical and confirmed neuropathy during the leg dependency period. However, the contribution of the neurogenic band decreased in the three other groups (Figure 3C; *p* = 0.07).

## 4. Discussion

After confirming findings from previous research that the microvascular response to leg dependency is impaired in confirmed neuropathy, this study explored the underlying flowmotion pattern, providing evidence that neurogenic activity increases during this blunted response in those with subclinical and confirmed neuropathy. 

The leg dependency protocol used in this study evoked a small VAR compared to venous congestion or standing protocols [11,12,25]. However, it induced a decrease in perfusion at both skin sites (i.e., the dorsal foot and the medial malleus). The decrease in skin blood perfusion was moderate at around 30% in the dorsal foot and 40% in the medial malleus. Nevertheless, this stimulus was able to discriminate between groups, revealing a blunted decrease in those with confirmed-neuropathy, as compared to controls and persons with T2DM; ultimately, suggesting a reduction in the vasoconstriction capacity of these participants. Importantly, the adjustments made for age did not change the result as previously reported in healthy persons [26]. Inflammatory status assessed by concentrations of fibrinogen and hs-CRP did not correlate with the findings in this study. Additionally, the concentrations of these biomarkers were not different between groups (not measured in controls), suggesting that inflammation is not responsible for the blunted vasoconstrictive response in confirmed neuropathy. To account for the intrinsic differences between persons with T2DM, who had higher foot temperature, and those without T2DM, the probe was not heated [27]. Thus, results were also adjusted for basal skin temperature. Skin temperature did not impact the VAR as already reported by Davison et al. [28]. Skin blood perfusion during the VAR did not return to basal levels at the end of the five-minute recording. It must be noted that this recording duration may not have been long enough. Indeed, a return to basal skin blood perfusion is achieved in 10 min in healthy persons [8].

The findings of this study, where the VAR was impaired in confirmed neuropathy but preserved in those with T2DM alone and in those with subclinical neuropathy, were not in accordance with the previous studies. Indeed, most studies have reported an altered response in those with diabetes. However, when looking at these studies in greater detail, some included persons with T2DM, with or without neuropathy in the same group [29]. Furthermore, patients with foot ulcers or older participants were also included [6,9,11]. In this present study, neuropathy was carefully defined as either subclinical or confirmed using recommendations from the Toronto consensus for clinical studies [19]. Additionally, participants in the obesity group were matched by gender and body mass index to those with T2DM, isolating the effect of T2DM. Therefore, the results of this study suggest that in subclinical neuropathy, the microvascular vasoconstrictive response is preserved before a progressive alteration is established in confirmed neuropathy.

Whether the underlying mechanisms of the VAR are similar in healthy persons and those with T2DM had not been explored previously. We reported an increase in total spectral power during the VAR at the medial malleus in all groups without changes in cardiac and respiratory absolute values. This increase of total power prompted the relative contribution of only low-frequency components to be discussed further.

Myogenic tone has usually been regarded as a major mediating factor in the VAR. Crandall et al. have demonstrated that the VAR was controlled by non α-adrenergic nerve fibers and myogenic tone, rather than by local sympathetic innervation [2]. Additionally, Fuji et al. identified a role of the Ca^2+^ and voltage-gated K^+^ channels [4]. Unexpectedly, activity in the myogenic band decreased during the VAR at both skin sites in all groups. However, this result was in accordance with findings from Silva et al. in healthy persons [3]. As the VAR is an immediate reflex, the myogenic component may be activated quickly during the first seconds of leg dependency to protect the capillaries from a substantial increase in hydrostatic pressure. Its involvement may then be decreased for the remainder of the evaluation, the extra six minutes necessary to obtain valid wavelet analysis [30]. Alternatively, this reduction in the contribution from the myogenic band might be explained by the substantial increase in the contribution to skin blood perfusion from the endothelial bands, which might override the myogenic mechanisms; reflecting the autoregulation from the vascular smooth muscle cells [13]. Considering this collectively, there may be cross-talk between each spectral component with compensatory phenomena in each band coming to the forefront according to the microcirculatory demands.

The two endothelial bands increased in all groups and across both of the skin sites. Indeed, this finding in persons with T2DM extended upon results in healthy persons [3]. These increases may be related to the release of vasodilator substances to increase blood flow, progressively restoring skin blood perfusion to basal conditions. 

Finally, there was no time or group effect in the neurogenic band at either skin site. However, a trend for a group by time interaction was noted at the medial malleus (*p* = 0.07), indicating a decrease in the neurogenic contribution to skin blood perfusion during the VAR in controls, in those with obesity and in those with T2DM, but an increase in subclinical and confirmed neuropathy. This blunted neurogenic contribution to skin blood perfusion during the VAR may be explained by the short period of sympathetic activation with the rise in transmural pressure after leg dependency, followed by a withdrawal throughout the sustained dependency period [3]. This is the first study to show different responses between neuropathy groups and others showing a dysregulation of sympathetic function in neuropathy. Indeed, the confirmed neuropathy slightly increased the activity within the neurogenic band, while the increase was more profound in subclinical neuropathy. This may be due to an over activity in the residual nerves of those with subclinical neuropathy, before total loss. A higher contribution in the neurogenic band may have allowed those with subclinical neuropathy to maintain a normal VAR.

There are several limitations to the present study. First, this study was conducted on hospitalized patients with T2DM and poor glycemic control. Therefore, the validity of these results may not be extrapolated to patients with well-controlled diabetes. Second, skin perfusion was assessed on non-glabrous skin at both sites. As the regulation of skin blood perfusion in non-glabrous and glabrous skin is different, these results might not extend to glabrous skin. Third, LDF recordings were assessed on the right foot only, for technical reasons. A randomization of the foot being assessed would have allowed for the assumption that the two limbs respond in the same way, especially since peripheral neuropathy is known to be symmetrical [31]. Finally, an evaluation of leg edema or swelling during lowering period might have brought other outcomes to the altered response in T2DM with neuropathy [8].

## 5. Conclusions

In conclusion, this study confirmed a blunted vascular response during the VAR in non-glabrous skin in T2DM with confirmed neuropathy but a preserved VAR response in T2DM with subclinical neuropathy. Our study provides new insights into the mechanisms that may contribute to this impairment. Overall, we demonstrated an alteration of the neurogenic response during VAR in patients with T2DM with neuropathy. Remarkably, we noted an hyper-activity of neurogenic component during VAR in early stage of neuropathy (i.e., subclinical) while response was significantly deteriorated in confirmed neuropathy. It is of clinical relevance for the prevention and treatment of foot ulcers to better understand the microcirculatory abnormalities in diabetic patients. Further research using more sensitive signal analyses might provide complementary insights into changes in skin blood perfusion mediated by the VAR.

## Figures and Tables

**Table 1:** Clinical characteristics of the participants.

	Controls (n = 16)	Obesity (n = 22)	T2DM (n = 15)	T2DM with Subclinical-DPN (n = 13)	T2DM with Confirmed-DPN (n = 16)
Women/Men	10/6	13/9	7/8	3/10	5/11
Age (years)	57.5 ± 1.9	53.9 ± 1.2	53.4 ± 1.9	59.9 ± 2.0	59.9 ± 1.5 ^#,‡^
BMI (Kg/m^2^)	23.5 ± 0.4	32.0 ± 0.8 ***	30.2 ± 1.3 ***	29.0 ± 1.0 ***	33.2 ± 1.5 ***
SBP (mmHg)	124.8 ± 4.0	127.9 ± 4.0	125.5 ± 2.8	128.5 ± 2.8	126.3 ± 4.0
DBP (mmHg)	75.7 ± 2.9	81.2 ± 2.7	79.4 ± 2.9	79.5 ± 3.3	76.1 ± 2.4
HR rest (bpm)	70.0 ± 3.7	60.9 ± 1.3	75.2 ± 3.2 ^###^	72.3 ± 2.7 ^###^	74.9 ± 2.9 ^###^
HbA1c (%)	-	5.4 ± 0.1	8.8 ± 0.3 ^###^	8.7 ± 0.3 ^###^	8.4 ± 0.2 ^###^
hs-CRP (mg/L)		3.1 ± 0.7	3.4 ± 0.8	3.2 ± 1.9	4.0 ± 1.0
Fibrinogen (g/L)		3.7 ± 0.1	3.8 ± 0.2	3.8 ± 0.2	4.3 ± 0.2
Diabetes duration (years)	-	-	12.7 ± 2.1	14.5 ± 2.1	14.3 ± 2.3
Retinopathy	-	-	5	3	4
Nephropathy	-	-	7	4	6
CAN	-	-	2	3	4
Medications					
OHM	-	-	6	6	4
Insulin injection	-	-	1	1	3
OHM + insulin	-	-	6	6	9
Anti-hypertensive	-	-	7	8	14
Dyslipidaemia	-	-	9	8	12
Neuropathic pain	-	-	1	0	1

Data shown as mean ± SEM. *** *p* < 0.001 vs. Controls; ^#^ *p* < 0.05 vs. Obesity; ^###^ *p* < 0.001 vs. Obesity, ^‡^ *p* < 0.05 vs. T2DM. DPN, diabetic peripheral neuropathy; T2DM, type 2 diabetes mellitus; BMI, body mass index; SBP, systolic blood pressure; DBP, diastolic blood pressure; HR, heart rate; HbA1c, glycated haemoglobin; hs-CRP, high sensitivity C-reactive protein; CAN, cardiac autonomic neuropathy; OHM, oral hypoglycaemic medication.

**Table 2:** Microcirculation variables at rest and during the venoarteriolar reflex in each group.

	Controls	Obesity	T2DM	T2DM with Subclinical-DPN	T2DM with Confirmed-DPN	GLM *p*-Value
Basal skin blood perfusion (PU)	10.3 ± 1.8	10.4 ± 1.5	10.4 ± 1.3	8.6 ± 1.0	9.1 ± 1.0	ns
VAR_MIN_ (PU)	4.8 ± 0.6	5.2 ± 0.4	5.2 ± 0.5	5.2 ± 0.6	5.6 ± 0.5	ns
VAR_MIN_ (%)	−44.7 ± 5.6	−39.9 ± 4.7	−44.8 ± 5.5	−36.2 ± 5.6	−35.4 ± 3.0	ns
T-VAR_MIN_ (sec)	143.4 ± 24.4	126.9 ± 17.5	102.9 ± 19.8	92.1 ± 19.4	84.6 ± 16.3	ns

Data shown as mean ± SEM. ns, non-significant; GLM, General linear model; DPN, diabetic peripheral neuropathy; T2DM, type 2 diabetes mellitus.

## Data Availability

The data presented in this study are available on request from the corresponding author. The data are not publicly available due to technical restrictions.

## Supplementary Materials

The following are available online at https://www.mdpi.com/article/10.3390/biology10040333/s1. Figure S1: Skin blood perfusion kinetics during leg dependency at the dorsal foot in each group. Figure S2: The relative contribution of each component at rest and during leg dependency at the dorsal foot.
