# Supplementary material for: Mechanisms of Venoarteriolar Reflex in Type 2 Diabetes with or without Peripheral Neuropathy
Source: Biology (Basel). 2021 Apr 15;10(4):333. doi: 10.3390/biology10040333 (PMC8071175; doi:10.3390/biology10040333)
Supplement: Supplementary file 1 [file biology-10-00333-s001.zip › biology-1178975-supplementary.pdf]

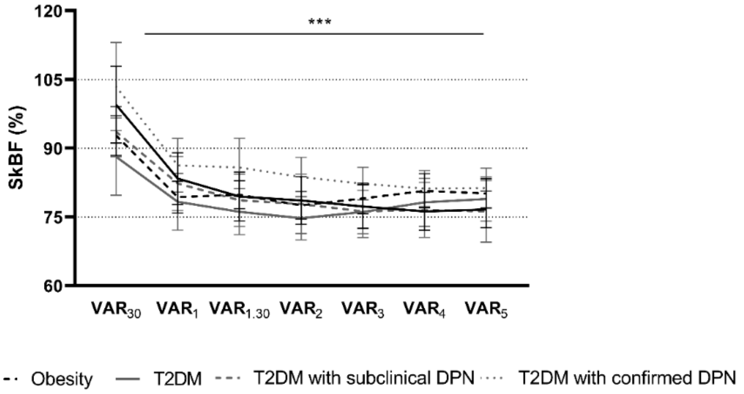

Figure S1. Skin blood perfusion kinetics during foot lowering at the dorsal foot in each group. *** Time effect $p < 0.001$; Group effect: $p = 0.09$; interaction: $p = 1$. DPN denotes diabetic peripheral neuropathy; T2DM, type 2 diabetes mellitus; SkBF, skin blood flow.

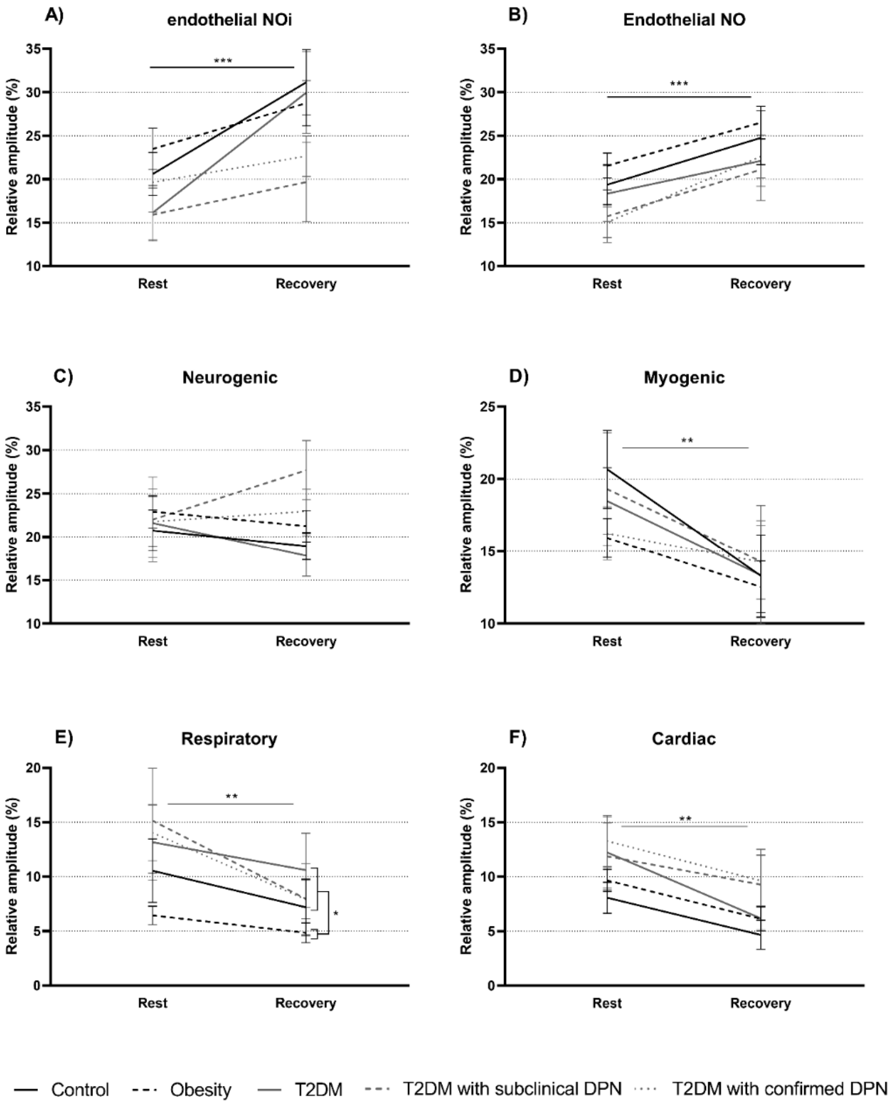

Figure S2. The relative contribution of each component at rest and during foot lowering at the dorsal foot : endothelial NO-independent (**A**), endothelial NO-dependent (**B**), neurogenic (**C**), myogenic (**D**), respiratory (**E**) and cardiac (**F**) band. ***Time effect $p < 0.001$; **Time effect $p < 0.01$; * Group effect T2DM vs Controls and Obesity $p < 0.05$. DPN, diabetic peripheral neuropathy; T2DM, type 2 diabetes mellitus.
